# Adaptation to Skew Distortions of Natural Scenes and Retinal Specificity of Its Aftereffects

**DOI:** 10.3389/fpsyg.2017.01158

**Published:** 2017-07-13

**Authors:** Selam W. Habtegiorgis, Katharina Rifai, Markus Lappe, Siegfried Wahl

**Affiliations:** ^1^Institute for Ophthalmic Research, University of Tuebingen Tuebingen, Germany; ^2^Institute of Psychology, University of Muenster Muenster, Germany

**Keywords:** visual adaptation, spatial distortions, translation invariance, natural vision, adaptation aftereffects

## Abstract

Image skew is one of the prominent distortions that exist in optical elements, such as in spectacle lenses. The present study evaluates adaptation to image skew in dynamic natural images. Moreover, the cortical levels involved in skew coding were probed using retinal specificity of skew adaptation aftereffects. Left and right skewed natural image sequences were shown to observers as adapting stimuli. The point of subjective equality (PSE), i.e., the skew amplitude in simple geometrical patterns that is perceived to be unskewed, was used to quantify the aftereffect of each adapting skew direction. The PSE, in a two-alternative forced choice paradigm, shifted toward the adapting skew direction. Moreover, significant adaptation aftereffects were obtained not only at adapted, but also at non-adapted retinal locations during fixation. Skew adaptation information was transferred partially to non-adapted retinal locations. Thus, adaptation to skewed natural scenes induces coordinated plasticity in lower and higher cortical areas of the visual pathway.

## Introduction

Spatial geometrical distortions often occur as artifacts in optical devices used in everyday life, such as spectacles and displays. Distortions alter many spatial features of a visual scene, including position information, form, or optic flow (Welch, 1969; Epstein, 1972; Vlaskamp et al., 2009). They cause loss of visual constancy and disrupt stable visual perception (Welch, 1978).

Adaptation is a mechanism by which the visual system adjusts its response to stabilize perception when features are altered in the visual world (Clifford et al., 2007; Webster, 2015). The visual system modifies its response when exposed to geometrical distortion of scenes (Regan and Hamstra, 1992; Suzuki et al., 2005). Robust visual adaptation to geometric distortion of image size magnification induced by meridional magnifying lenses was previously demonstrated (Adams et al., 2001; Yehezkel et al., 2010). Yet, the corresponding cortical origin of the plasticity during vision in distorted dynamic natural visual inputs is not well-explored. Specifically, contribution of higher cortical areas for the robust plasticity in distorted dynamic natural visual input has not been assessed.

Adaptation induces plasticity along the hierarchical visual stream; from lower to higher cortical levels (Helson, 1964; Webster, 2011). Lower level cortical areas, such as V1, are retinotopically organized and the aftereffects of their plasticity can only be observed at the specific adapted retinal location (Hubel and Wiesel, 1968; Van Essen and Anderson, 1995; Clifford et al., 2000; Dickinson et al., 2010). The receptive field size of neurons increases at higher levels enabling them to integrate the information over a wide range of the visual field (Gattass et al., 2005). Thus, aftereffects originating from plasticity of higher cortical neurons can be transferred across different retinal locations. Retinal position invariance therefore allows identification of higher level distortion encoding mechanisms (Suzuki and Cavanagh, 1998; Zhao and Chubb, 2001; Afraz and Cavanagh, 2008).

Usually, the input to the visual system is rapidly changing natural image content. Natural images contain a great variety of visual features such as spatial frequency, luminance, contrast, orientation, texture, color, or optic flow signals (Dong and Atick, 1995; Billock et al., 2001; Bex and Makous, 2002; Betsch et al., 2004; Bex et al., 2005, 2007, 2009). Optically induced image modifications such as astigmatic blur and distortions of spectacles alter multiple features of the natural world; and the visual system adapts to them (Adams et al., 2001; Sawides et al., 2010; Yehezkel et al., 2010; Vinas et al., 2012, 2013). Accordingly, plasticity of the visual system to optical modification of the dynamic natural environment might involve coordinated responses of several neural populations tuned to different stimuli features. These coordinated responses might not always be revealed by adaptation responses of specific groups of neurons to a selected stimulus feature under controlled experiments (Gallant et al., 1998; Ringach et al., 2002; David et al., 2004; Felsen and Dan, 2005). Specifically, the study of the visual system's natural adaptation behavior, probably involving a diversity of neural populations, benefits from using scenes that mimic the dynamics of the natural environment.

In the present study, visual adaptation to skew distortion was studied systematically in psychophysical experiments with natural scenes. Adaptation to image skew is a prominent challenge in todays' natural visual world. Progressive additional lenses (PALs) are common spectacles inducing such a distortion as an inevitable artifact (Meister and Fisher, 2008; Barbero and Portilla, 2015). Some novice PAL wearers report prolonged discomfort to progressive additional lenses due to swim effect and spatial disorientations (Sheedy and Andre, 2005). In this group of PAL wearers, lack of adaptation to image skew is a candidate source of the reported visual discomfort. The image skew is assumed to be one of the sources of visual discomfort and adaptation difficulty experienced by novice PAL wearers.

**Figure 1:**
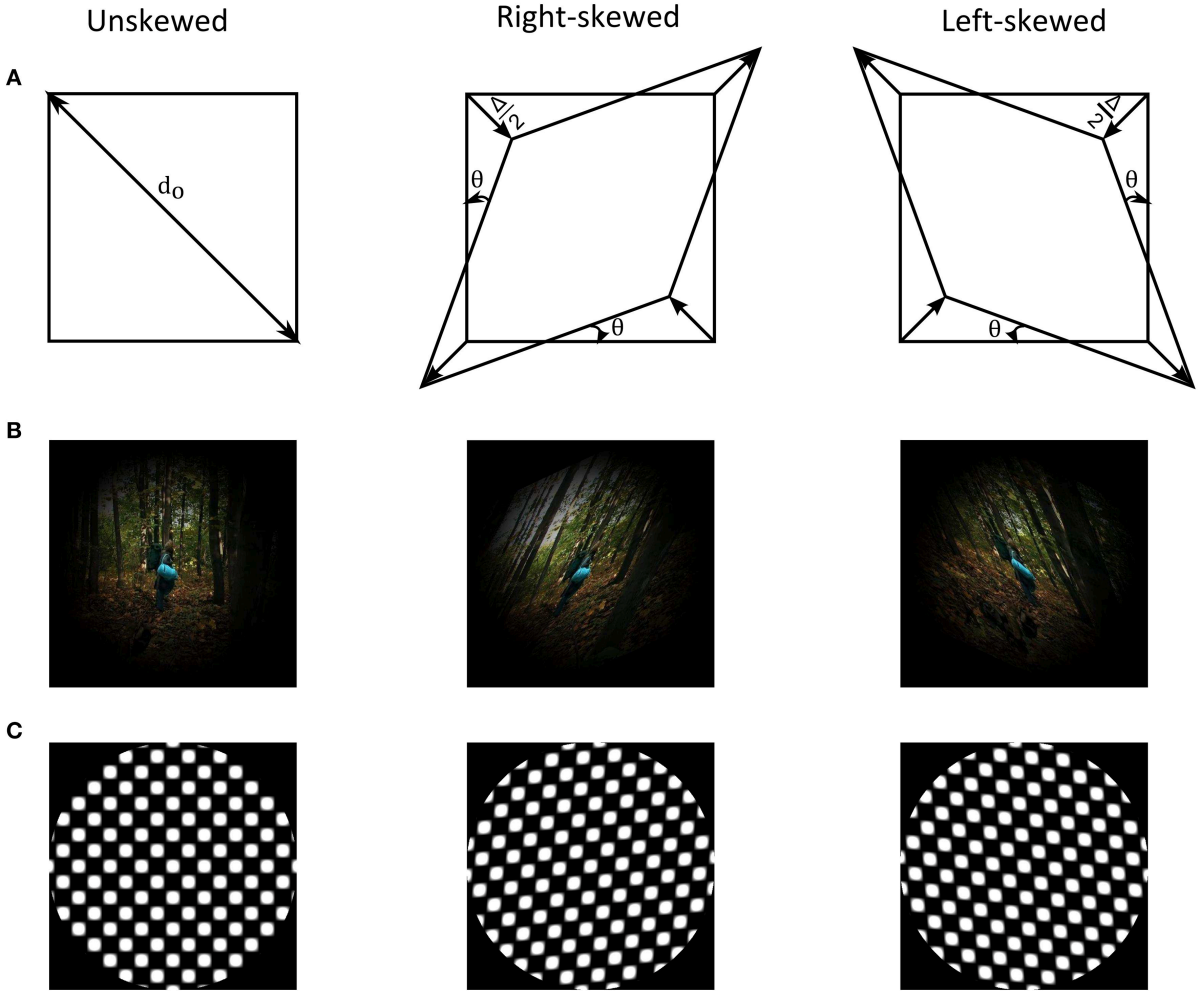
**(A)** Illustration of skew geometrical distortion. **(B)** Example of a natural image from the adaptation image sequence together with its skewed counterparts that are weighted by a Hanning window. **(C)** Example of an unskewed test plaid checkerboard stimulus with its skewed counterparts.

Skew geometrical distortion shears and unequally magnifies images in oblique meridians (Fannin and Grosvenor, 1987). Figure 1 shows an illustration of the distortion as well as examples of natural images and geometrical patterns. The shear and the magnification alters orientation and spatial frequency statistics of natural images, respectively. These alterations activate and change responses of lower level cortical areas (Field, 1987; Bao and Engel, 2012; Snowden et al., 2012; Dekel and Sagi, 2015). The oblique magnification of image skew additionally modifies global form features, such as dimensional symmetry, of scenes. Encoding of dimensional symmetry as a global feature in simple geometrical patterns was suggested in prior studies (Regan and Hamstra, 1992; Suzuki et al., 2005). Thus, image skew in natural scenes could activate neurons at various cortical levels along the visual pathway.

In the first experiment, adaptation to skew distortion in natural scenes was demonstrated. Subsequently, in a second experiment, retinal location specificity of the skew adaptation aftereffects was used to probe cortical levels involved in skew coding. Partial position invariance of the skew adaptation aftereffect was found. Aftereffects occurred at adapted and non-adapted retinal locations. Thus, visual adaptation to a skewed natural environment shows a new class of global adaptation to distortions mediated by coordinated plasticity of higher and lower cortical areas.

## Experiment 1: adaptation to image skew in natural scenes

The purpose of this experiment was to investigate if exposure to skewed natural scenes changes visual perception.

### Materials and methods

Adaptation was induced by presenting skewed natural image sequences to observers. Adaptation to skew was evaluated in a two-alternative forced choice paradigm. Psychometric curves of skew perception were computed after exposure to right and left skewed natural stimuli. The skew magnitude in the test stimuli that was perceived to be unskewed was used to measure adaptation effects.

#### Study approval

The study was approved by the Ethics Committee of the Medical Faculty of the Eberhard Karls University of Tübingen and the University Hospital.

#### Observers

Ten observers, aged 18–40 years, participated in this psychophysical experiment. All but one observer were naïve about the purpose of the study. While taking part, all observers had normal or corrected to normal vision. Observers gave their informed written consent, in adherence to the Declaration of Helsinki, prior to participating in the experiment.

#### Set-up

The psychophysical procedure was designed and stimuli were generated in Matlab (Mathworks, MA, USA) using the PsychToolbox routines (Brainard, 1997) on an apple computer (Apple, USA). An LCD monitor (Benq corporate, USA) was used to display the stimuli at a screen resolution of 1,920 × 1,080 pixels (square pixels, with 0.31 mm pixel pitch) and a screen refresh rate of 60 Hz.

The viewing distance was maintained at 57 cm using a chin and head rest. At this observation distance, the whole screen subtended a visual angle (VA) of 55° horizontally, and 33° vertically. The stimuli were presented at the center of the screen in an otherwise completely darkened room and subtended a VA of 20° both vertically and horizontally. Left, right and space keys of a keyboard were used to collect observer's responses during adaptation aftereffect measurements.

#### Stimuli

The adapting stimuli were skewed natural image sequences (Figure 1B). Natural images were taken from an open source movie (Baumann, 2010). Each natural image, sized 1,280 × 720 pixels, was skewed in Matlab (Mathworks, MA, USA) by remapping the pixel positions of the undistorted image, *x* and *y*, into new distorted pixel positions, *x*_*d*_ and *y*_*d*_, using the geometrical transformation matrix, *M*, Equation (1) and (2).

(1) $$\left(\begin{array}{c} x_{d}\\ y_{d} \end{array}\right)=M*\;\left(\begin{array}{c} x\\ y \end{array}\right)$$

(2) $$M=\left(\begin{array}{c} 1\;\;\;\;-tan\theta \\ -tan\theta \;\;\;\;\;1\;\; \end{array}\right)$$

In Equation (2), θ is the shear angle in horizontal and vertical directions.

The inner 650 × 650 pixels of each distorted image were used cropping out the rest to remove sheared edges. Thus, the images subtended a VA of 20° × 20° in horizontal and vertical directions. Boundary effects were further reduced by applying a Hanning window as a weighting function (see Equation 3) (Harris, 1978). This weighting function, *w*, had the same size as each image, 650 × 650 pixels. Its intensity was 1 at the center and decreased radially outwards reaching zero at the boundaries (Equation 3).

(3) $$w(r)=\cos^{2}\left(\frac{\;\pi }{N}\;r\right)$$

In Equation (3), *r* is the radial distance of the pixel position from the center of the image and *N* was set to be equal to the image dimension, i.e., 650 pixels.

Two groups of adapting stimuli, containing oppositely skewed image sequences (left-skewed at θ = +25° and right skewed at θ = −25°), were prepared. The image content was identical in both distorted image sequences. Each adapting image sequence consisted of 18,000 image frames. During adaptation, these image sequences were rendered at a rate of 20 frames per second, thus each image was refreshed three times when presented on the 60 Hz display.

Plaid checkerboards, of the same dimension as the adapting images and distorted by different skew amplitude, were used as test stimuli to measure the adaptation aftereffect (Figure 1C). The skewed plaid checkerboard images were constructed by superimposing identical contrast sinusoidal gratings oriented at −45° to the right and +45° to the left. The dimensions of the squares' diagonals in the plaid correspond to the spatial wavelengths of the component gratings. The skew was induced by varying the wavelengths of the two component gratings, as in Equation (4).

(4) $$d_{right}=\;d_{o}-\Delta ,\;\;d_{left}=\;d_{o}+\Delta$$

*d*_*right*_ and *d*_*left*_ are the dimensions of the right and the left diagonals of the plaid and corresponds to the wavelengths of the left and right oriented component gratings, respectively. Δ, in pixels, is the wavelength variation parameter to induce the skew in the plaids. When unskewed, i.e., Δ = 0, the diagonals of the plaid squares have equal dimensions of *d*_*o*_ = 40 pixels subtending a VA of 1.24°. Non-zero Δ stretches the plaid diagonally and shears the zero-crossings of the squares in the plaid. Positive Δ corresponds to left skewed plaid and negative Δ to the right skewed plaid.

The skew amplitude was quantified by the magnification in oblique directions which is induced by either geometrically shearing or varying the diagonal dimensions, as presented in Equation (5).

(5) $$Skew_{amplitude\left(\Delta \backslash \theta \right)}=1-\frac{d_{right}}{d_{left}}=1-\frac{d_{o}-\Delta }{d_{o}+\Delta }=1-\frac{1-\tan\theta }{1+\tan\theta }$$

#### Procedure

Before taking part in the psychophysical experiment, observers were informed about the procedure and trained on how to respond to the test stimuli using a keyboard. Viewing was monocular.

Adaptation was tested alternately to the oppositely skewed image sequences, first to the left-skewed then to the right-skewed natural image sequences. The adaptation aftereffect was tested after each adaptation using the method of constant stimuli. Ten amplitudes of skew were used for the test stimuli. Ten responses were recorded for each skew amplitude. In total, 100 responses were recorded to compute the psychometric curves of each adapting skew direction.

Observers fixated at the center of the screen. Each skewed adapting image sequence was shown first for 3 min to induce adaptation and then for 15 s after each test stimulus presentation to top up the adaptation. Test stimuli were presented for 2 s. The skew amplitude of the test stimuli was in a randomized order. After each test stimulus presentation, observers had to report whether the skew direction of the plaid checkerboard was to the right or to the left by pressing the right or the left key of a keyboard, respectively.

#### Statistical data analysis

At each skew amplitude of the test stimuli, the percentage of leftward responses was computed. The percentage of leftward skew responses as a function of skew amplitude of test stimuli was then fitted with a cumulative Gaussian using Psignifit 4.0 software (asymptotes set free but assumed to be equal) (Schutt et al., 2016). The point of subjective equality (PSE), i.e., the skew amplitude at 50 percent of leftward responses indicated the skew amplitude that was perceived as undistorted. The size of the adaptation aftereffect, ΔPSE, was evaluated as the difference between the PSE of the left-skew and the right-skew adaptations. The overall aftereffect was computed by averaging the ΔPSEs from all the observers. A paired sample *t*-test was conducted on the ΔPSEs to evaluate the significance of the shift in perception due to exposure to skewed scenes.

#### Result

**Figure 2:**
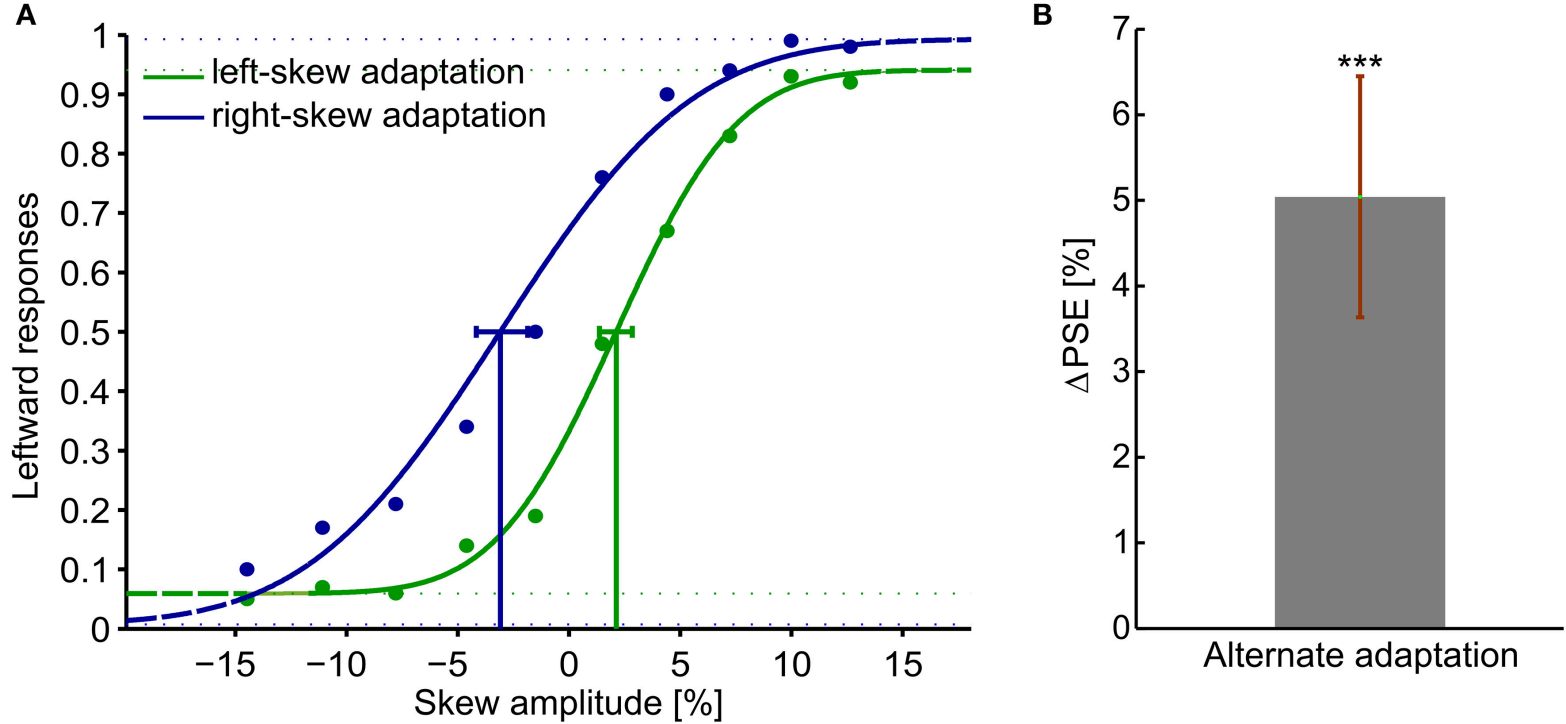
Adaptation aftereffects in response to skewed natural image sequences. Adaptation aftereffect is estimated by the PSE, the skew amplitude at which observers respond equally likely that the test plaid checkerboards are skewed to the right and to the left. **(A)** psychometric functions of the average response of all the observers. The gaussian fitted function, data points and the confidence intervals at PSE are shown in green for the left-skew adaptation aftereffects and in blue for the right skew adaptation aftereffects. **(B)** The overall Δ**PSE** of all the 10 observers after left and right skew adaptation. The error bar shows the standard error. ^***^t-Test result of *p* < 0.05.

Figure 2A presents psychometric functions of the average response of all the observers. The percentage of leftward responses as function of the skew amplitude is shown. A negative skew amplitude corresponds to a right-skewed and a positive value to a left-skewed plaid checkerboard. All observers showed significant aftereffects with psychometric functions resembling the overall data. Thus, the PSE shifted to the direction of the adapting skew. After adaptation to right-skewed natural stimuli, observers perceived right-skewed plaid checkerboard as undistorted and vice versa. The magnitude of the PSE shift induced by left and right skew adaptations relative to the PSE measured in the training trials before any adaptation had comparable sizes (*p* > 0.05). The ΔPSEs measured after alternate adaptation to the right-skewed and left-skewed stimuli are significantly different form zero, *p*-value < 0.01. Overall subjects' average ΔPSE is shown in Figure 2B.

In sum, an adaptation aftereffect was obtained after adaptation to skewed natural dynamic image sequences. The visual system continuously recalibrated its response after alternate adaptation to oppositely skewed dynamic natural stimuli. Thus, a robust and stimulus-independent plasticity of the visual system to skew distortions was demonstrated.

## Experiment 2: retinal transfer of skew adaptation aftereffect

In this psychophysical experiment, we assessed whether higher level cortical areas contribute to the skew adaptation. Retinotopic and non-retinotopic adaptation conditions were tested wherein adaptation aftereffect was examined at adapted and non-adapted retinal locations during fixation, respectively.

### Materials and methods

As in experiment 1, skewed adaptation was induced by showing distorted natural image sequences to the observers. Aftereffects were then measured using an adjustment procedure wherein observers had control over the skew angle of the test stimulus to adjust it until it is perceived undistorted.

All the materials and methods were the same as in experiment 1, except the changes noted below.

#### Observers

Ten participants partook in this psychophysical experiment. All but one participants were naïve about the purpose of the study. Participants gave their informed written consent, in adherence to the Declaration of Helsinki, prior to participating in the experiment.

#### Set-up

The stimuli were displayed on a ViewPixx/3D monitor at a resolution of 1,920 × 1,080 pixels and vertical refresh rate of 100 Hz in an otherwise darkened room. A chin and head rest was used to maintain the viewing distance of 60 cm at which the display subtended VA of 47° horizontally by 27° vertically. The lateral position of the stimuli was controlled gaze contingently by recording the right eye's position in real time at 1 kHz sample rate with the Eyelink 1,000 Plus eye tracker (SR Research, Ltd., Ontario, Canada) and the Eyelink toolbox (Cornelissen et al., 2002). Participants' response was recorded using the left, right and space keys of a keyboard.

#### Stimuli

Adapting stimuli were natural image sequences as in experiment 1. Each image in the sequence subtended 13° × 13° of VA at zero eccentricity. The test stimulus was a white cross image on a black background skewed at different angles by the transformation matrix in Equation (2). The corresponding skew amplitude in the test stimulus at each skew angle, *Skew*_*amplitude*_(θ), was computed using Equation (5). The cross test stimulus was used since it is easily detectable in the periphery. At the 0 skew amplitude, the test stimuli subtended 9° × 9° of VA when viewed at zero eccentricity.

#### Procedure

Participants were informed about the procedure and trained on how to adjust a skewed cross until they perceive it undistorted while viewing was monocular and peripheral.

**Figure 3:**
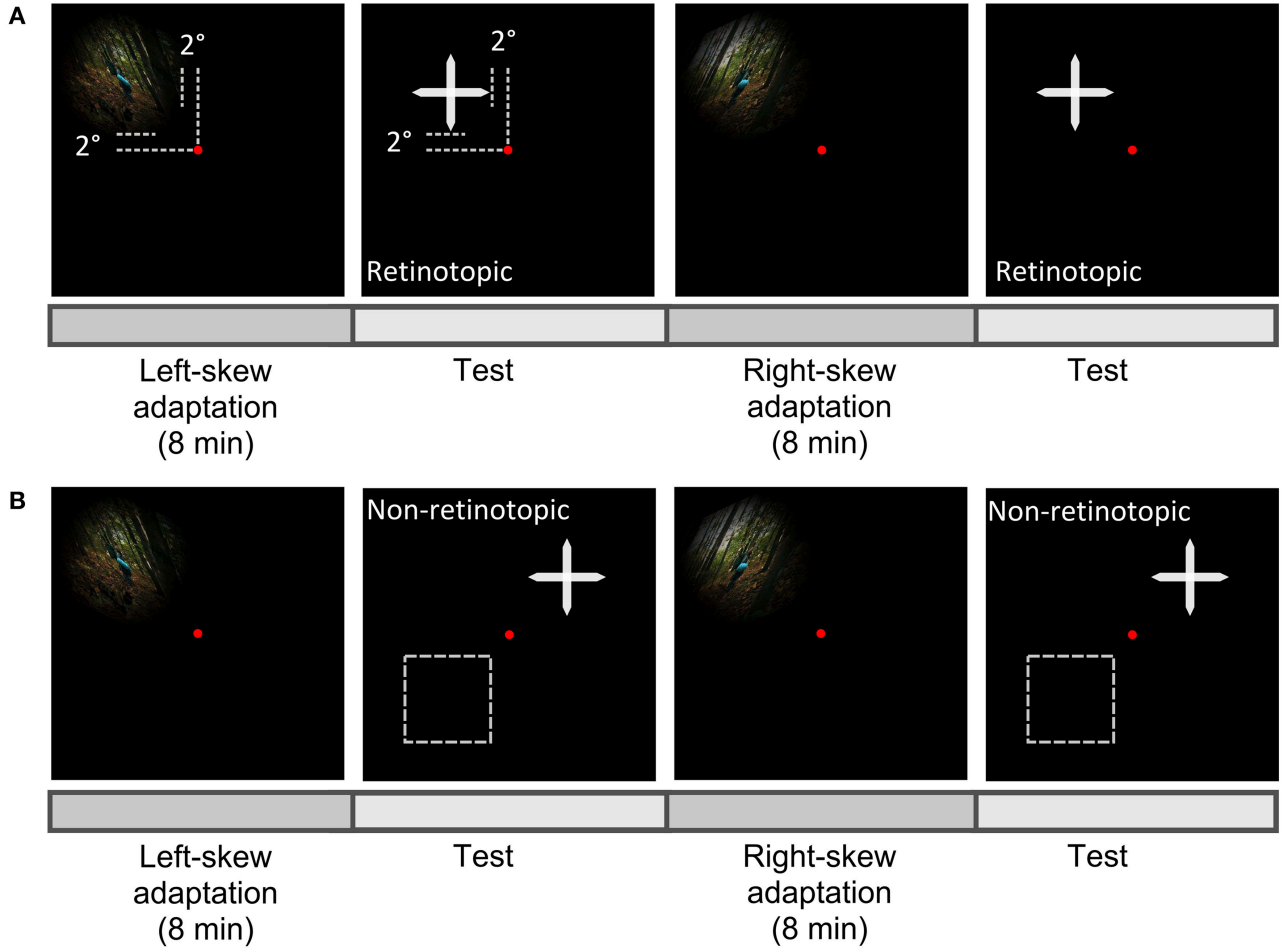
Scheme of experiment paradigm. Observers fixated centrally at the red dot and the aftereffect was measured in the upper-left visual field as the adapter in the retinotopic condition **(A)**, or at a new location either in the upper-right or lower-left (the broken lines) visual field in the non-retinotopic condition **(B)**.

In both conditions, the retinotopic and the non-retinotopic condition, the perceptual shifts were inspected after alternate exposure to the oppositely skewed image sequences (Figure 3). To induce adaptation, observers watched left and right skewed image sequences each lasting 8 min, each followed by a test sequence. Aftereffects of each adapting skewed stimuli were assessed by an adjustment procedure. After each adaptation, the cross image skewed at a random angle between 3° and –3°, was presented on the screen. The observers' task was to adjust the skewed cross until it was perceived to be unskewed. The left and the right keys of a keyboard were used to increase or decrease the skew angle with a step size of 0.5° and the space key was used to confirm the perception of the undistorted cross. The skew angle, at which the cross was perceived as undistorted, was used to compute the skew amplitude at the PSE with Equation (5). Fifteen trials of adjustment were performed in each aftereffect measurement step.

In both conditions, skewed adapting stimuli were presented in the upper-left visual field at 2°From the fixation dot (Figure 3). Gaze was fixed at the center of the screen throughout the experiment. Aftereffects were tested at adapted and non-adapted locations in the retinotopic and non-retinotopic condition, respectively (Figure 1). Performance asymmetries for visual tasks were previously reported between upper-lower as well as left-right visual fields (Karim and Kojima, 2010; Abrams et al., 2012; Matthews and Welch, 2015). To reduce any bias from these asymmetries in the non-retinotopic condition, the transfer of adaptation was measured across left-right visual fields for half of the observers and across upper-lower visual fields for the other half.

Observers' gaze was tracked in real time during the whole adaptation measurement procedure. Whenever observers made an eye movement toward the stimuli, the stimuli vanished with a delay of less than 40 ms. This assured the presentation of the stimulus in the desired retinal location. Thus, in the non-retinotopic condition, the adapting and test stimuli did not overlap.

#### Statistical data analysis

For each observer, the PSEs of the left and right skew adaptations in the 15 adjustment trials were binned into three; i.e., trial 1—trial 5 in bin 1, trial 6—trial 10 in bin 2 and trial 11—trial 15 in bin 3 (Equation 6).

(6) $${\text{PSE}}_{ave}(bin_{i})=\frac{1}{5}\;\sum_{i\;=\;1}^{3}\sum_{j\;=\;5i-4}^{5i}\text{PSE}(trial_{j})$$

The magnitude of the aftereffect, ΔPSE, was computed by subtracting the averaged PSEs of the left and right skew adaptations in each bin.

(7) $$\Delta \text{ PSE}\;\left(bin_{i}\right)=PSE_{ave\_left-skew}\left(bin_{i}\right)-PSE_{ave\_right-skew}\left(bin_{i}\right)$$

For the non-retinotopic condition, the transferred adaptation to the new locations was quantified by Δ*PSE* in the first bin as percentage of the corresponding retinotopic first bin Δ*PSE*.

(8) $$Transfered\;adaptation=\frac{\Delta PSE{\left(bin_{1}\right)}_{non-retinotopic}}{\Delta PSE{\left(bin_{1}\right)}_{retinotopic}}*100$$

The overall average of the aftereffects' magnitude and the transfer was calculated. Paired-sample *t*-tests were performed to estimate significant differences of the overall averages from zero. An ANOVA was conducted to evaluate the influence of retinotopy and bin number, i.e. relative test time, on the magnitude of the aftereffect.

#### Result

**Figure 4:**
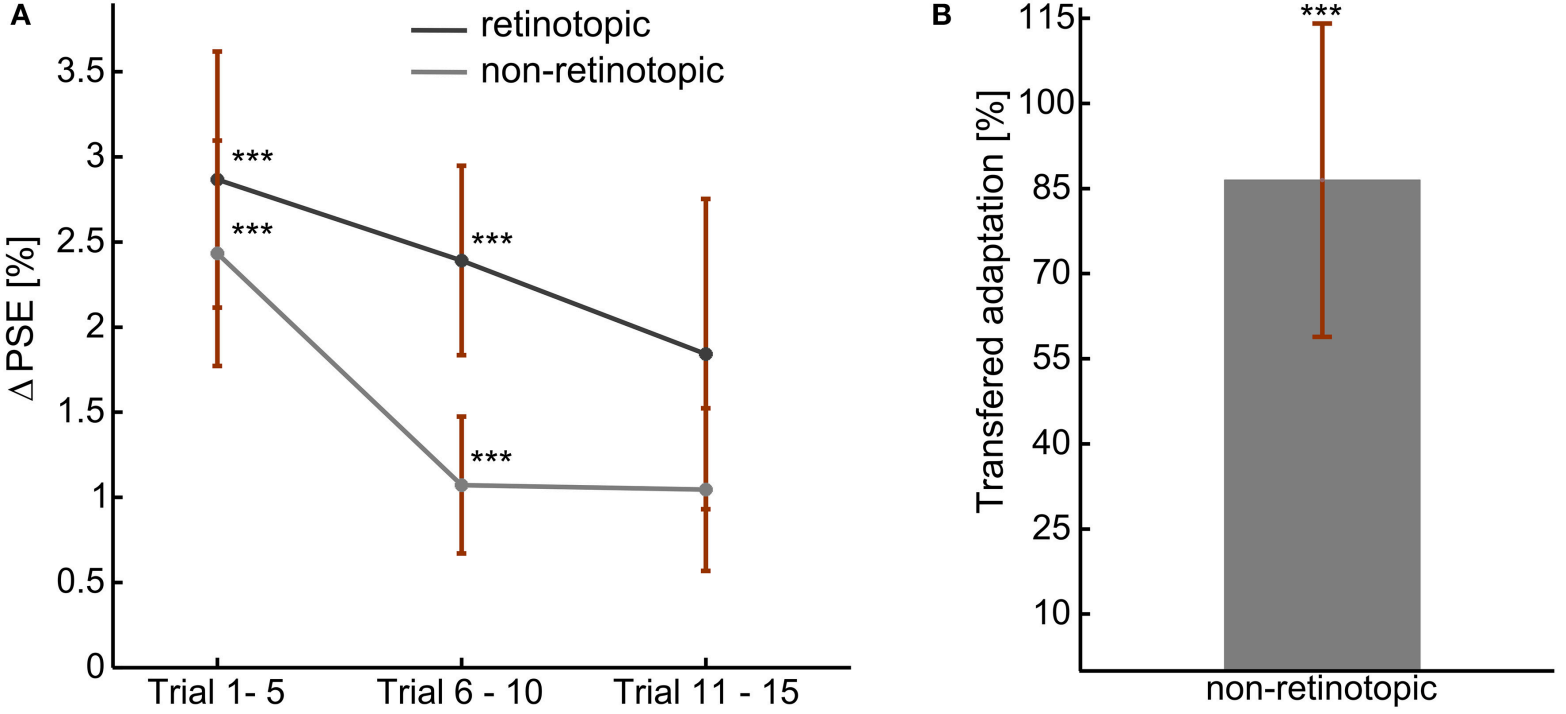
Skew adaptation aftereffects during fixed gaze, at retinotopic, and non-retinotopic locations. **(A)** Overall Aftereffect magnitude. **(B)** Overall transfer of adaptation: non-retinotopic aftereffects' magnitude as percentage of retinotopic aftereffect. The error bars show the standard errors. ^***^t-Test result of *p* < 0.05.

The adaptation aftereffects of all the observers in each adaptation measurement condition is presented in Figure 4. A positive shift in PSE confirmed the aftereffect demonstrated in experiment 1 (Figure 4A). Moreover, as in experiment 1, the left and right skew adaptations induced comparable magnitude of PSE shift relative to the training PSE (*p* > 0.05). Aftereffect magnitudes in the first two bins were significantly different from zero for both retinotopic (*p* (bin 1) < 0.01, *p* (bin 2) < 0.01) and non-retinotopic (*p* (bin 1) < 0.01, *p* (bin 2) < 0.04) conditions. Albeit there was no significant main effect of the bin number, the magnitude showed a decreasing trend through the test trials [ANOVA: *F*_(59, 2)_ = 1.75, *p* > 0.2]. The aftereffects of the two conditions are not significantly different from one another [ANOVA, *F*_(59, 1)_ = 1.53, *p* > 0.2]. Thus, skew adaptation aftereffects at retinotopic and non-retinotopic locations had comparable sizes. Figure 4B shows the amount of adaptation transferred to the new retinal location. 86.5% of the adaptation was significantly transferred to an un-adapted retinal location (*p* < 0.02).

Therefore, at a fixed gaze, adaptation aftereffects of image skew can be observed at adapted and non-adapted retinal locations.

## Discussion

In the present study, we demonstrated visual plasticity to skew distortions in natural scenes in two psychophysical experiments. In the first experiment, the general perceptual effect of exposure to image skew in natural scenes was revealed. The PSE of observers was shifting in the direction of the adapting skew direction, i.e., alternate adaptation to a left (positive) then a right (negative) skewed natural stimuli resulted in positive shift of the PSE. Thus, the visual system continuously recalibrated its response in correlation with the direction of the adapting skew irrespective of the stimuli image content. In the second experiment, to reveal the plasticity of higher cortical areas, retinal specificity of the skew adaptation aftereffect was inspected during fixation. At retinotopic and non-retinotopic locations, a positive shift of the PSE was observed as an adaptation aftereffect. More importantly, 86.5% of the adaptation effect was transferred to a new retinal location. Accordingly, during fixation, part of the skew adaptation effect was independent of the retinal location of the stimuli. This can be achieved only if neural mechanisms with large receptive field sizes partook in encoding of the skew information (Leopold et al., 2001; Zhao and Chubb, 2001; Afraz and Cavanagh, 2008). Therefore, shift invariance of skew adaptation indicates plasticity of extrastriate higher level cortical areas in addition to retinotopically organized lower level cortical areas.

Aftereffects originate from response changes in specific neural populations tuned to the adapting stimuli attributes (Roach et al., 2008; Webster, 2015). Thus, adjustments in neural substrates processing the altered features in the skewed natural images lead to the measured aftereffect. Moreover, adaptation to attribute rich natural scenes activates several neural populations along the different visual cortical areas and might involve their dynamic interactions as well as coordinated responses (Berkley et al., 1994; Dakin et al., 1999; Smith et al., 2001; Poirier and Frost, 2005; Pavan et al., 2013).

Lower level contrast and contour orientation processing mechanisms in early stages of the visual system stream, such as simple cells in V1, exhibit fully retinotopic receptive fields (Hubel and Wiesel, 1968; Wilkinson et al., 1998; Zhao et al., 2011; Dickinson and Badcock, 2013). These early stage visual processing mechanisms are well-tuned to orientation distribution of the contrast energy in natural scenes (Field, 1987; van Hateren and van der Schaaf, 1998). They adjust their response depending on orientation statistics of the natural environment (Bao and Engel, 2012; Dekel and Sagi, 2015). Skew distortion also alters the orientation statistics of images resulting in high contrast energy in the oblique direction which changes responses of the retinotopic orientation selective mechanisms. The induced lower level plasticity conceivably contributes to the adaptation aftereffect which was not transferred to the new retinal location.

In the visual processing stream, complex shape, and motion processing mechanisms in higher cortical areas feature large receptive field sizes (Van Essen and Anderson, 1995; Gattass et al., 2005; Suzuki et al., 2005; Mather et al., 2008). Skew alters global form features, such as angles or points of high curvature, and dimensional symmetries. Area V2 contributes to angle information extraction by a linear combination of orientation selective V1 cells' outputs (Hegdé and Van Essen, 2000; Boynton and Hegdé, 2004; Ito and Komatsu, 2004). Angle discrimination and judgment of the visual system is highly dependent on global geometry of the object containing them (Kennedy et al., 2006, 2008). Curvatures and orientation flows of real and illusory contours also activate three dimensional and complex shape processing mechanisms in extra-striate cortical areas (Pasupathy and Connor, 1999, 2001; Li et al., 2008; Filangieri and Li, 2009). Furthermore, higher cortical areas such as V4 and IT, encode complex geometric features including dimensional symmetry from global configurations of the oriented contours (Regan and Hamstra, 1992; Wilson and Wilkinson, 2002; Suzuki et al., 2005; Loffler, 2008). Furthermore, even direct encoding of symmetry variations, including oblique magnification by skew, as a global feature was previously suggested (Regan and Hamstra, 1992; Suzuki et al., 2005). Thus, the plasticity in the aforementioned higher level complex shape processing mechanisms potentially contributed to the reported shift invariant component of skew adaptation aftereffect. Further studies with series of dedicated experiments can reveal the exact origin of the higher level skew encoding from natural scenes.

Optical modifications of the visual world, such as astigmatic blur and distortions, are daily life constraints in spectacle wearers (Fannin and Grosvenor, 1987; Meister and Fisher, 2008). Proper habituation is essential in challenging visual situations, such as vision in elderly, where mobility can be affected, e.g., in stepping and falling (Johnson et al., 2007). Here, we showed adaptation to geometric distortions with ecological image content. Our methodology provides a tool to address the contribution of adaptation to distortion in real life scenarios, e.g., habituation to PALs.

In sum, the visual system is able to extract skew information from the dynamic natural environment and induce a robust stimulus-independent adaptation. Furthermore, plasticity of both lower and higher cortical areas account for skew adaptation in dynamic natural environment.

## Author contributions

SH and KR designed the study. SH conducted the experiment, collected, and analyzed the data. All authors interpreted the data, contributed intellectual content to the manuscript, and approved the final submission.

### Conflict of interest statement

The authors declare that the research was conducted in the absence of any commercial or financial relationships that could be construed as a potential conflict of interest.
